# Copper Tube Pitting in Santa Fe Municipal Water Caused by Microbial Induced Corrosion

**DOI:** 10.3390/ma7064321

**Published:** 2014-06-05

**Authors:** Thomas D. Burleigh, Casey G. Gierke, Narjes Fredj, Penelope J. Boston

**Affiliations:** 1 Materials & Metallurgical Engineering Department, New Mexico Tech, Socorro, NM 87801, USA; E-Mail: nfredj@nmt.edu; 2 Earth & Environmental Sciences, New Mexico Tech, Socorro, NM 87801, USA; E-Mails: caseygierke@gmail.com (C.G.G.); pboston@nmt.edu (P.J.B.)

**Keywords:** copper, pitting, microbes, SEM, biocorrosion, microbiologically influenced corrosion, MIC, microbial induced corrosion, tap water, municipal water

## Abstract

Many copper water lines for municipal drinking water in Santa Fe, New Mexico USA, have developed pinhole leaks. The pitting matches the description of Type I pitting of copper, which has historically been attributed to water chemistry and to contaminants on the copper tubing surface. However, more recent studies attribute copper pitting to microbial induced corrosion (MIC). In order to test for microbes, the copper tubing was fixed in hexamethyldisilazane (HMDS), then the tops of the corrosion mounds were broken open, and the interior of the corrosion pits were examined with scanning electron microscopy (SEM). The analysis found that microbes resembling actinobacteria were deep inside the pits and wedged between the crystallographic planes of the corroded copper grains. The presence of actinobacteria confirms the possibility that the cause of this pitting corrosion was MIC. This observation provides better understanding and new methods for preventing the pitting of copper tubing in municipal water.

## 1. Introduction

This study was initiated upon receipt of copper tubing from several different residential cold water lines that had developed pinhole leaks after fifteen years of transporting municipal water. There are currently hundreds of papers written on the subject of the pitting corrosion of copper tubing in municipal drinking water. Unfortunately, as reported by Lytle and Nadagouda (2010) [1], “Despite past research, pitting corrosion of copper plumbing is still poorly understood, unpredictable and difficult to remediate.” Farooqui (2009) reports that there is, “…incomplete understanding of the pinhole phenomenon” [2].

The types of copper pitting are often categorized into three (or four) groups; Type 1 pitting occurs in cold water lines, Type 2 occurs in hot water lines, and Type 3 occurs in Sweden [3,4,5]. Type 1.5 is similar to Type 1 but it stains positive in the Periodic Acid Shift test for polysaccharide, which indicates the presence of microbes [5]. What is in common for Types 1, 2, and 3 pitting is that the copper pits are covered with a copper oxide membrane, and above the membrane is a porous mound or cap. Beyond these similarities, their appearances diverge greatly. The mounds are often composed of malachite [4], while other times they are sulfates [1]. The pit cavities may be concentrated in either chlorides [1,6] or sulfides for Type 3 [5]. 

In the published scientific literature, there are three proposed causes for the copper pitting, and they can be categorized as due to: (1) the water chemistry; (2) the copper surface contaminants; (3) microbial induced corrosion, or a combination of the above.

The water chemistry that leads to pitting corrosion of copper has been a subject of intense research. Sarver reported that heavily chlorinated, high pH, low alkalinity water will pit copper [7]. They found that pitting was greatly reduced by dosing the water with high levels of orthophosphates or silicates. Ha *et al.* [8] and Ha and Scully [9] used artificial pits and electrochemical potentials to study pit growth in different composition electrolytes. Ha *et al.* [9] found the highest pit growth rates were in water containing sulfates. 

Francis [3] stated that water associated with pitting corrosion was generally from deep bore holes (wells), low in organics, and with certain levels of sulfate, chloride, oxygen and pH. 

Surface contaminants have also been linked to the pitting corrosion. The 1950s work by Campbell [10] showed the relation of the pitting to a residual carbonaceous film on the surface of annealed copper tubing. The carbon film on the copper surface was residue from the high temperature anneal of the drawing-lubricant-coated tubing [10]. Francis [3] reported that properly cleaned copper tubes were less likely to pit. Oliphant and Jönsson [4] ascribed the pitting to a carbon surface film from the bright anneal process, and to water quality, and that Type I pitting did not occur above 40 °C. 

Many researchers have ascribed the copper-pitting problem to microbial induced corrosion (MIC). Wagner (1997) did a very thorough literature review with 118 references that attributed pitting of copper water lines to bacteria. Wagner concluded that the most promising preventive measure was by controlling the water chemistry; modulating the alkalinity while optimizing the chloride/sulfate ratio [11]. Reyes (2008) analyzed the microbes that were contributing to the high copper content (blue water) [12]. He provided an extensive review of the types of microbes found on biofilms on copper water lines. Reyes reported the biofilms on the houses he examined contained *Variovorax* sp [12].

Cantor (2006) [13] reported on an investigation of pinhole leaks in copper pipes in the furthest reaches and dead ends of the Brown Deer Water Utility distribution system. The interior of the corroded pipes had corrosion pits beneath green mounds. After ruling out manufacturing flaws, installation flaws, soil interactions, stray electrical currents, and water chemistry, the pipes were sent to the Wisconsin State Lab of Hygiene. The microbiologists there used nucleic material stains and microscopy and found microbe-laced biofilms in the pitted areas and corrosion debris of the corroded pipes. After a new chlorination booster station was placed in service, the pinhole leaks decreased along with a decrease in the heterotrophic plate count [HPC] of microbes in the water [13].

Geesey *et al.* [5] (1993) reported on the many unusual types of pitting corrosion of copper tubes in potable water systems. Although microbes were considered as a possible cause of the pitting, he stated that there had been no definitive proof. In order to prevent MIC, Geesey recommended that the water temperature be maintained below 25 °C for cold water and above 55 °C for hot water [5]. 

Labuda *et al.* [14] (2012) described MIC of a domestic copper hot-water system. He reported that the internal surfaces of the copper tube exhibited tan-colored, groove-like areas that were separated by dark brown-colored islands that had much less wall thinning. The grooves were perpendicular to the direction of flow, and the corrosion pitting showed severe undercutting (tunneling) of the copper. Noteworthy, the tan or dark brown surfaces occurred in hot water service, and were different than the green corrosion products observed in cold water service by Oliphant [4].

The irony of microbial induced corrosion of copper is that for centuries copper sheeting has been used for anti-fouling to prevent the growth of barnacles on ocean-going vessels [15]. Copper is also well-known for its anti-microbial properties and its ability to kill pathogenic microorganisms [16]. However to a microbiologist, this dual nature of copper is neither unusual nor surprising.

The following study describes the scanning electron microscopic analysis of the corrosion pits in copper tubing that show the presence of microbes.

## 2. Results and Discussion

### 2.1. Optical Microscopy

**Figure 1:**
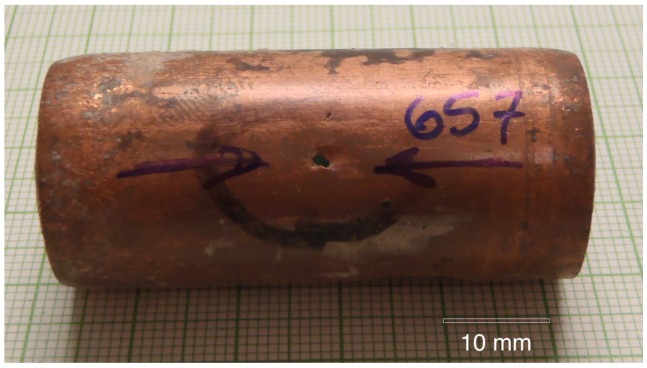
A typical pinhole leak in the copper tubing.

**Figure 2:**
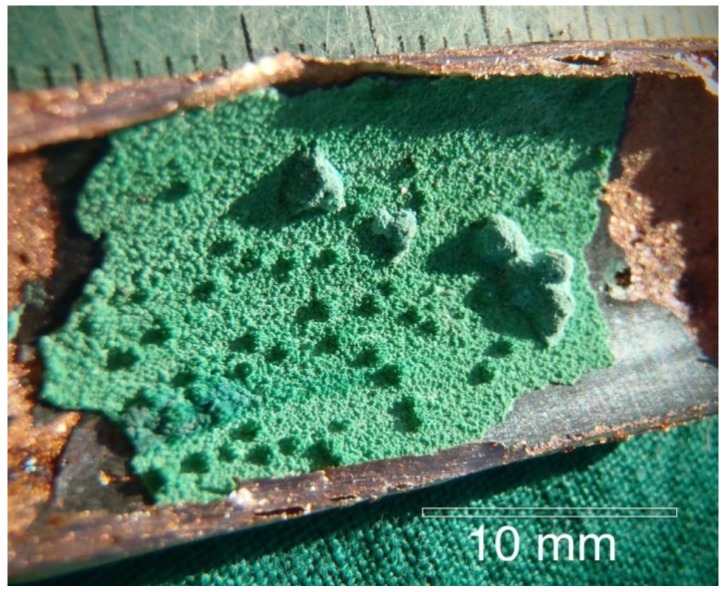
The malachite mounds found on the interior of the copper tubing.

Copper tubing #657 with a typical pinhole leak is shown in Figure 1. The high velocity water exiting the pinhole washed away all of the features that were associated with the initiation of the pinhole leak. Therefore nearby regions of the tubing were examined in order to find corrosion pits that had not yet perforated the complete wall thickness. The interior of the tube was typically covered by a green layer (later identified as malachite) with various sized mounds (or caps) as shown in Figure 2. 

Examination of Figure 2 reveals four distinct regions. First are the large and small green mounds, ranging from several millimeters in width and height, to less than 1 mm in width and height. Second is the uniform green layer that is very brittle and easily spalls. Beneath the green layer is a third layer that is a black, thin, featureless copper oxide. When the black oxide was chipped off, the shiny, underlying copper surface was seen. (The XRD results for the green and black oxide layers are shown in the next section.) The hot-water copper surfaces that corroded by MIC according to Labuda [14] were completely different from the cold-water copper surfaces observed in this investigation.

### 2.2. X-ray Diffraction

**Figure 3:**
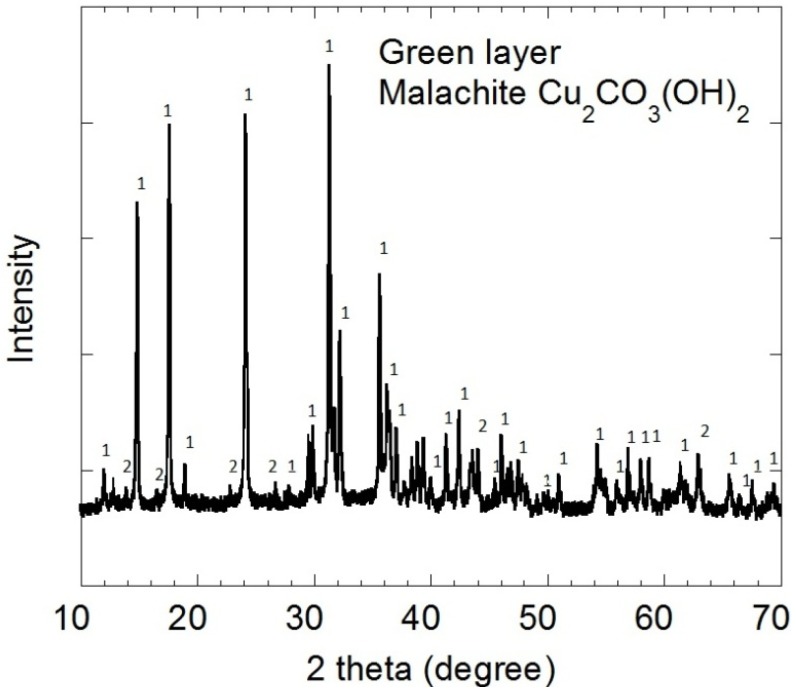
The XRD spectra for the green layer correspond primarily to malachite (peaks labeled “1”).

**Figure 4:**
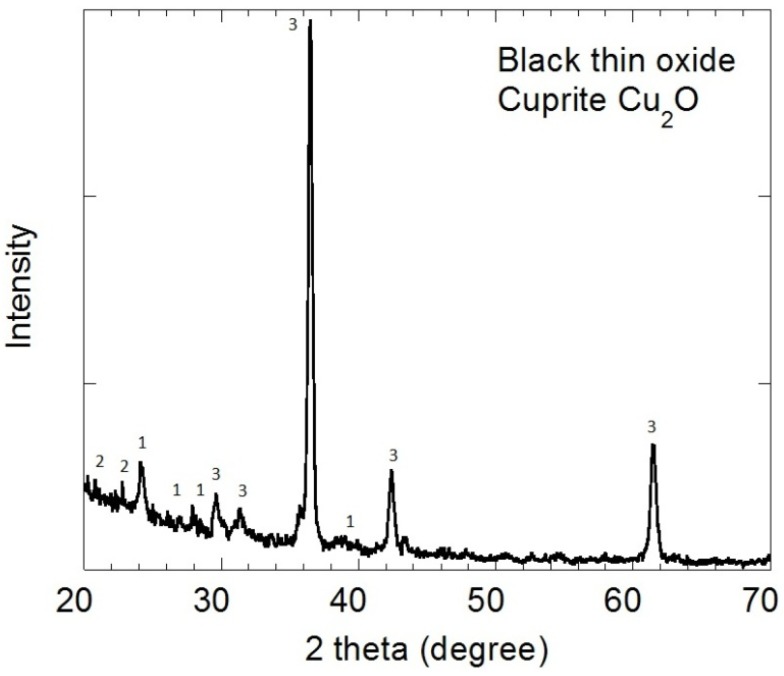
The XRD spectra for the black oxide layer correspond primarily to cuprite (peaks labeled “3”).

The surface oxides inside tubing #806 were characterized by X-ray diffraction (XRD) and glancing angle X-ray diffraction (GAXRD). The XRD spectra of the upper green layer in Figure 3 corresponds to malachite, Cu_2_CO_3_(OH)_2_, peaks labeled “1”. The GAXRD pattern for the underlying black oxide in Figure 4 corresponds to cuprite, Cu_2_O, peaks labeled “3”. There is trace malachite “1” on top of the cuprite “3” in Figure 4. The peaks labeled “2” are trace unknowns.

### 2.3. Scanning Electron Microscopy (SEM)

**Figure 5:**
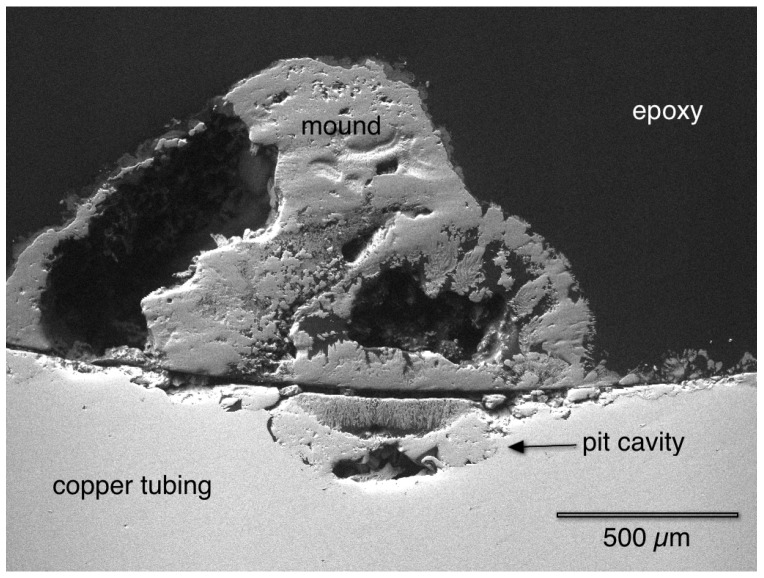
The cross-section of a mound shows that it was directly above a corrosion pit cavity.

Figure 5 shows the cross-section of a copper pipe that was polished to the middle of a mound. The large mound was not solid, but rather porous, and was located directly above the corrosion pit in the copper tubing. The Energy Dispersive Spectroscopy (EDS) analysis (not shown here) verified that the mound contained primarily copper (Cu), oxygen (O), and carbon (C), which is in agreement with malachite. The upper part of the mound contained trace sulfur (S) and inside the bottom of the pit, contained trace chloride (Cl), similar to previous reports [5]. 

**Figure 6:**
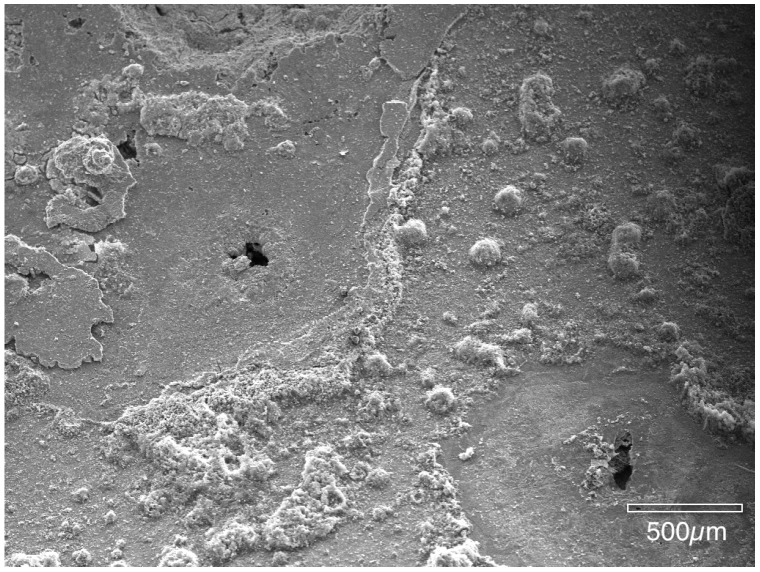
Once the mound was removed, the cuprite membrane shows small openings to the pit cavity.

**Figure 7:**
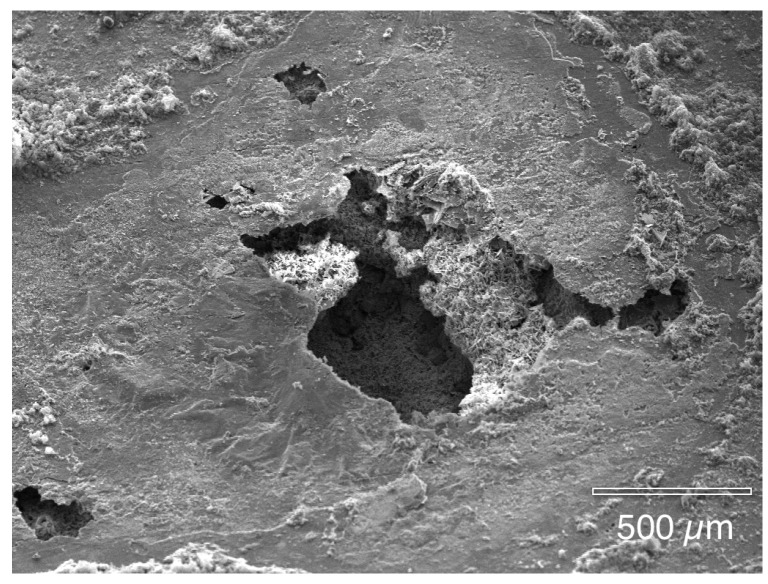
When the cuprite membrane was fractured, the larger underlying pit cavity was seen.

The next images show the interior surface of the copper tubing that was fixed in HMDS. Prior to examination on the SEM, the malachite mounds were broken open, platinum sputter-coated and then placed in the SEM. In this manner, the authors assure that all features found inside the pits had developed during water service and were not a result of sample preparation. Figure 6 and Figure 7 show the cuprite surface film after the overlying malachite mounds were removed. In Figure 6 there is a small opening connecting the upper mound to the lower pit cavity. Figure 7 shows the partially fractured membrane with the large underlying pit cavity. These observations are in agreement with the porous membrane model reported by previous authors [4,5,6].

**Figure 8:**
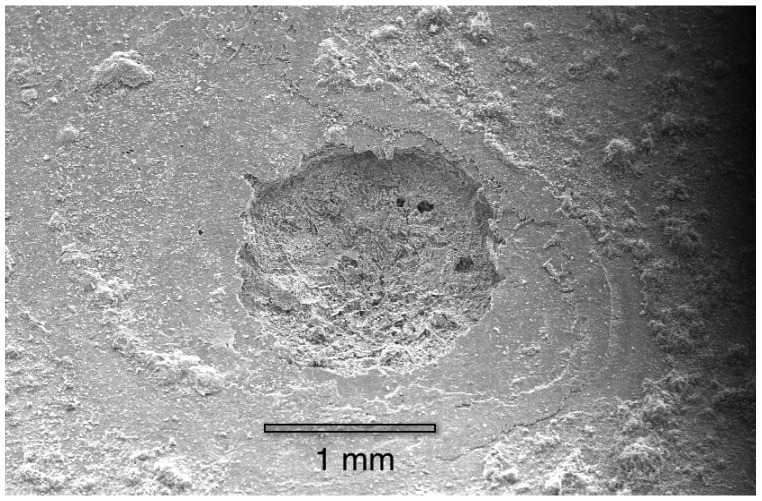
A corrosion pit, after both the malachite mound and cuprite membrane were removed.

Figure 8 shows a corrosion pit that was exposed by completely fracturing the fragile cuprite membrane by tapping the copper tube. 

**Figure 9:**
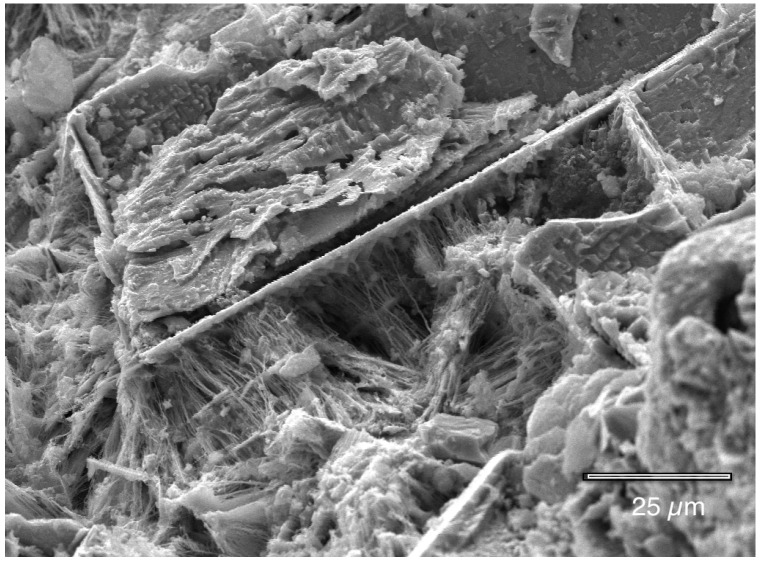
Microbial filaments deep inside a corrosion pit (hexamethyldisilazane (HMDS) fixation).

**Figure 10:**
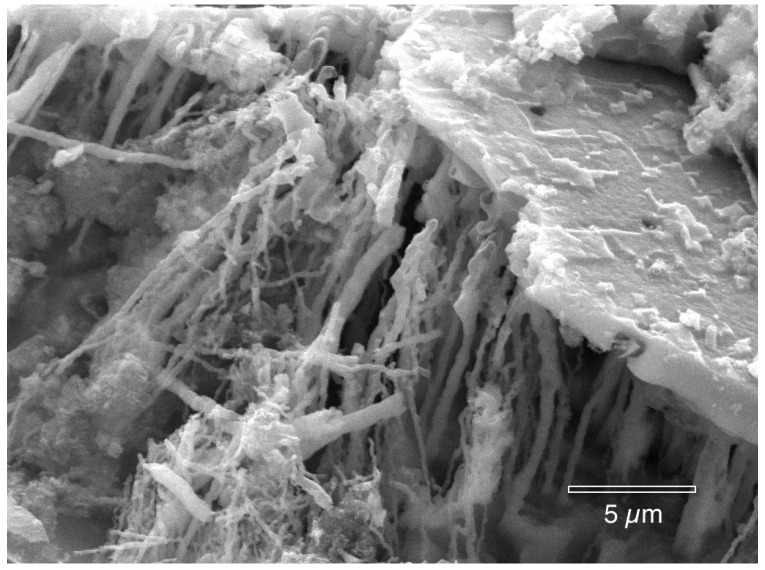
Microbial filaments deep inside a corrosion pit (HMDS fixation).

**Figure 11:**
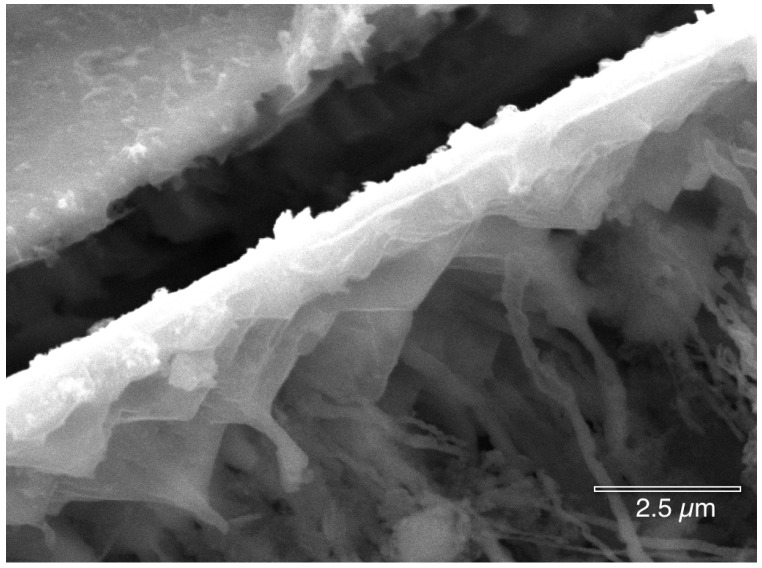
Microbial filaments attached to the copper inside a pit (HMDS fixation).

**Figure 12:**
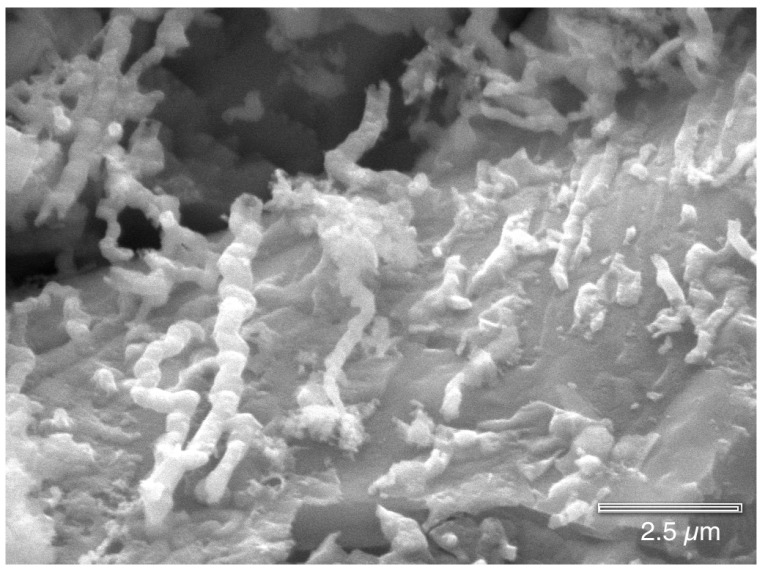
Clusters of microbes similar to Actinobacteria are deep inside a pit (HMDS fixation).

**Figure 13:**
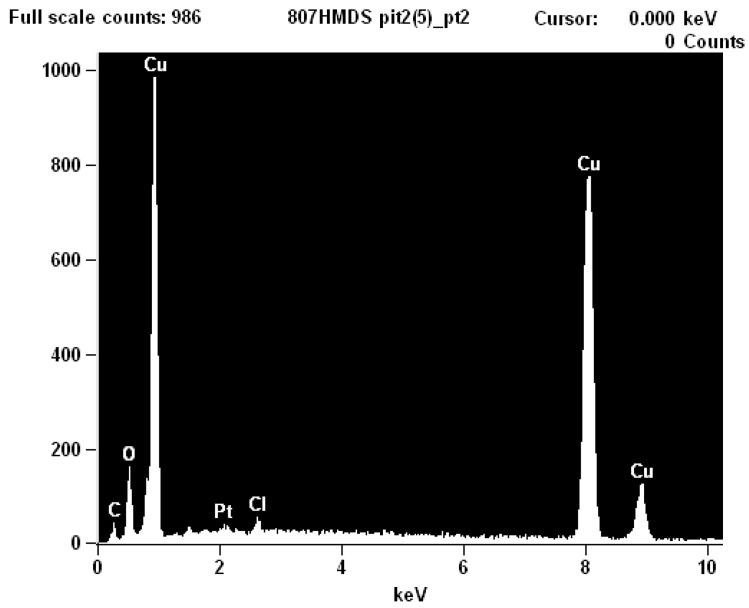
EDS (Energy Dispersive Spectroscopy) analysis of the microbes inside the pit shows only Cu, C, O and Cl.

**Figure 14:**
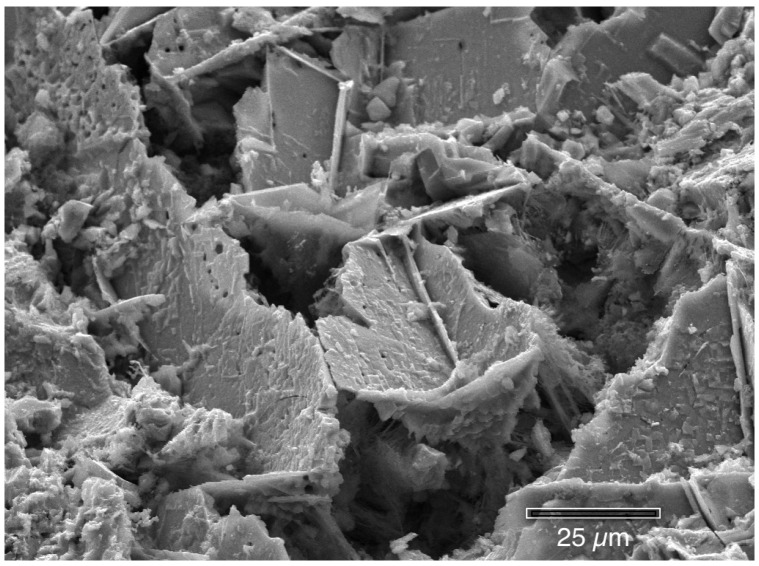
The copper crystallographic surfaces inside the pit (HMDS fixation).

The following photographs show the interiors of several different corrosion pits that were fixed in hexamethyldisilazane (HMDS). There are many features present. Figure 9, Figure 10, Figure 11, Figure 12, Figure 13, and Figure 14 show masses of filaments deep inside the corrosion pits of tubing #807. The filaments resemble some morphologies seen in the largest known phylum of bacteria, the Actinobacteria [17]. Many organisms in this very ancient group appear as chains of cells or spores, curled or spiral filaments, fuzzy mounds of cells, and many other unusual shapes and configurations [18], especially in the more extreme environments, like metal-rich (including copper) and subsurface environments [19,20]. Figure 9 and Figure 10 show the microbes filling the small cavities left behind after the dissolution of the copper surface. The tight geometry of this environment is ideal for the microbes because they are able to control the pH, the amount of free oxygen, and other chemical properties of their microenvironment.

The crystallographic pyramids in Figure 11 demonstrate how the bacteria attach to the surface, and protect their foothold from corrosion, while the rest of the surface is dissolved.

Figure 12 shows clusters of microbes, similar in appearance to Actinobacteria (to be discussed in the next section), across the interior of the copper pit surface.

EDS analysis (Figure 13) inside the pit consistently indicates only the presence of Cu, C, O and Cl, regardless of whether the beam was focused on the microbes or on a crystallographic surfaces. It was difficult to obtain a strong EDS signal inside the pit due to the sides of the pit absorbing the emitted X-rays. The crystallographic surfaces in Figure 14 show that the copper has been etched away due to either the microbial metabolism or the acidic nature of the pit. This surface topography provides many locations for the microbes and their biofilm to reside and to adhere. 

**Figure 15:**
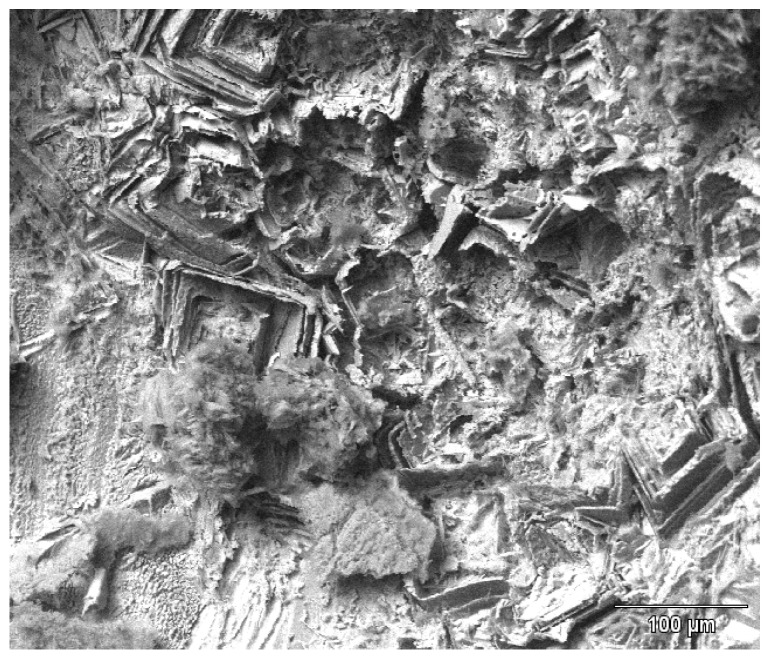
The copper crystallographic surfaces inside a pit (not fixed in HMDS).

**Figure 16:**
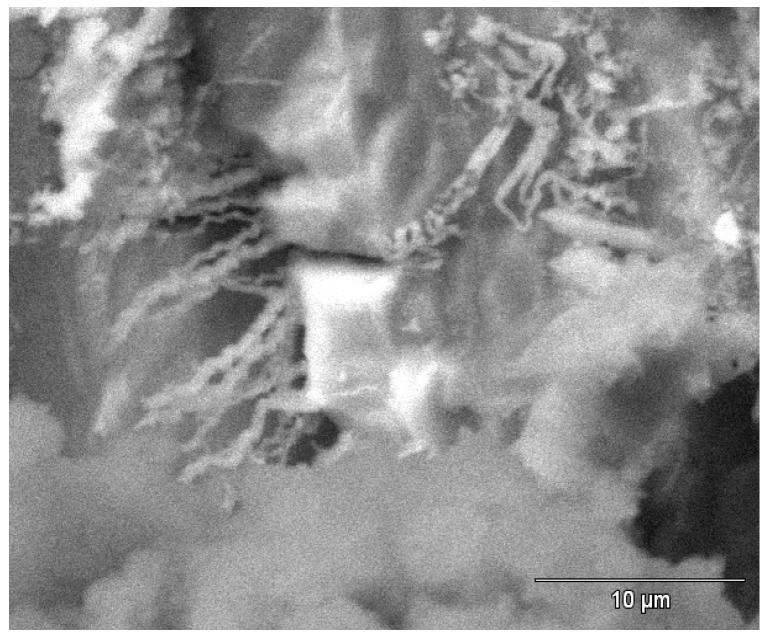
The micron-size filaments inside the crystallographic gaps (not fixed in HMDS).

Figure 15 and Figure 16 show tube #806 that was not fixed in the HMDS. Figure 15 shows crystallographic attack revealing the copper grains boundaries and the internal crystallographic planes. Figure 16 shows the one micron diameter filaments protruding from one copper grains. As shown in Figure 16, it is possible to find microbial evidence inside pits that were not fixed in HMDS, but the air-dried microbes tend to dehydrate and collapse, or explode in the vacuum or under the electron beam, and thus are not easily identifiable.

### 2.4. Discussion

The above SEM micrographs demonstrate that microbes are present deep inside corrosion pits in the copper tubing. Since the malachite shells (caps or mounds) and the cuprite membranes over the pits were intact until after drying, these microbes were present inside the pits while the copper tubes were in service, and are not laboratory artifacts. The microbes appear to be Actinobacteria based on morphological and ecological similarities to genetic and biochemical identifications of organisms in other copper-rich settings [19,20]. Although there have been many studies that have identified the bacteria on the surface of corroded water supply lines [11,12,13,21,22] this is the first known SEM investigation that not only has found the microbes deep inside the copper corrosion pits but also wedged inside crystallographic planes in the corroded copper. The microbes could be either passively involved in pitting (excreting an acid that dissolved the copper [23,24]) or actively using copper oxidation as a byproduct of energy acquisition [25] or even as a primary energy source [26] although that is typically associated with copper sulfides rather than elemental copper. 

The authors propose that the Actinobacteria were able to gain a foothold on the surface of the copper tubing due to the presence of residual organic films, such as extrusion lubricants or degreasing compounds. The residual films provided the organic carbon source necessary for establishment, and once established, microbial communities are able to derive enough carbon from trace carbon in water sources, or even from chemoautotrophic metabolism of other trace materials in the environment like low levels of carbonate. As copper tubing degradation proceeds, the pits begin to form and increasingly complex microbial communities thrive as copper dissolution creates confined pockets between crystallographic remnants. (The tight geometry of these pockets allows microbial communities to control their microenvironments, specifically, to lower the pH and alter redox conditions.) Simultaneous to the formation of the pits is the formation of a cuprite cover membrane and malachite mound, both of which further serve to help control the inner pit environment, by minimizing mixing with the bulk fluid. From this point, degradation continues until the exterior tube wall is breached and the pinhole leak forms, washing away evidence of the preceding events. 

The above conceptual model explains why the newer homes could experience copper pitting and leaks while older homes do not, despite having the same municipal water flowing through the copper tubing. The composition of the copper tubing has not changed, but it is probable that there have been changes in the composition of the extrusion lubricants or in the degreasing compounds that are used in the manufacturing of the copper tubing. Type I pitting of copper was first linked to residual, burnt-on lubricating oils [10]. It is possible that a new residual oil, lubricant, or degreasing compound is now providing the microbes with a carbon foothold on the copper tubing surface. Unfortunately, drawing lubricants are continually evolving over time, and copper tubing manufacturers are reluctant to disclose proprietary information about compositions.

Additional laboratory tests could determine the mechanism by which the microbes corrode the copper, and the identities of such organisms. Future work will include DNA analysis of the microbes, biological staining and epifluorescent microscopy of both cellular and biofilm materials, culturing the microbes in the laboratory to confirm their role in copper oxidation, and most importantly, determining what promotes or inhibits their growth. Electrochemical techniques may be used to non-destructively determine the rate of localized pitting.

The most pragmatic issue is how to prevent these microbes from causing pinhole leaks in copper tubing. The authors have explained how residual oil films could promote the establishment of these colonies. Previous studies have demonstrated that the role of water temperature is important [4,5] with microbes thriving only in a certain temperature range. The microbes may also be impaired or eradicated by applied potentials, chemicals (chlorination), biocides, radiation or ultraviolet light. 

## 3. Experimental Section

### 3.1. Study Sites

Homes in a residential neighborhood in Santa Fe, New Mexico, USA, have experienced over forty in-wall and under-slab leaks in the past four years. The age of the affected homes ranges from 15 to 20 years old, and 95% of the leaks have occurred in the domestic cold water lines, the majority of which occurred during the winter, often under floors heated by radiant floor heat. Older homes in the same or adjacent neighborhoods, receiving the same supply water, have not experienced the large number of leaks that these newer homes have experienced. 

The homes from which the tubes were taken all used City of Santa Fe municipal drinking water. The municipal water of Santa Fe is supplied by three sources; the City Well Field, the Canyon Road Plant, and the Buckman Well Field. These three sources are interconnected, so that municipal water at the homes could come from any one or any mixture of these three sources. Table 1 shows the reported water chemistry [27]. The pH of the water was near neutral, and there is a large range in ionic concentrations. Very little can be concluded based on this wide range of water chemistry. 

**Table 1:** Variations in the municipal water chemistry in 2010 for Santa Fe, NM.

Parameter	Low	High
Chloride (mg/L)	≤10	45
Sulfate (mg/L)	Not detected	75
Hardness (Ca & Mg) (mg/L)	90	263
pH	7.0	8.2

### 3.2. Copper Tube Sampling

Multiple copper tubes were examined, but only copper tubing that were corroded internally due to municipal water contact were included in this study. Sections were cut from the copper tubing near the original pinhole leaks. The pit cross-section was made by embedding the tube in epoxy, and then grinding down to the location of the pit. The cross-section was then sanded with progressively finer grits of SiC, to a final polish with 1 µm alumina slurry.

The copper tubing in all of the homes was manufactured according to the ASTM B88 Standard for Seamless Copper Water Tube (99.9% Cu + Ag).

### 3.3. Characterization Techniques

The samples were characterized by XRD and SEM. Crystallographic structure was obtained via the glancing angle X-ray diffraction (GAXRD) patterns using the X’Pert-PRO Diffractometer (PANalytical, Westborough, MA, USA) at the New Mexico Bureau of Geology & Mineral Resources. The XRD Diffractometer had a thin film attachment, unfiltered Cu-Kα radiation and a step size about 0.02°. The GAXRD had a glancing incidence angle of 0.8°. The peaks were identified using the Inorganic Crystal Structure Database (FIZ-Karlsruhe, 2012). For SEM studies, the copper tubes were platinum sputter-coated prior to examination on the Hitachi S-3200N scanning electron microscope (SEM) (Hitachi Ltd., Tokyo, Japan) at an accelerating voltage of 25 kV. Elemental analysis was performed using characteristic X-rays (EDS).

### 3.4. Fixation in Hexamethyldisilazane (HMDS)

Through the drying process, exposure to the high vacuum and to the electron beam in the SEM, the fragile microbe bodies are generally destroyed. In order to preserve the microbes for high vacuum SEM examination, tubing #807 was prepared by a hexamethyldisilazane (HMDS) fixation process similar to the process described by Dekker [28]. The process preserves the cell bodies of microbes, but it does not preserve the biofilm in which the microbes live. Tubing #807 was removed from the cold water line and kept wet or submersed in municipal water until it was placed in a 2.5% gluteraldehyde solution. The gluteraldehyde solution was used to cross-link polymers in the microorganism cell bodies, and stop metabolism. The copper tube sample was next progressively dehydrated by soaking it in increasingly concentrated ethanol solutions. The water was high purity molecular grade water and the ethanol was 200 proof ethyl alcohol. The ethanol was next replaced by soaking it in progressively more concentrated solutions of HMDS, and finally allowed to air dry in a desiccator. The chemical formula for hexamethyldisilazane (HMDS) is C_6_H_19_NSi_2_. The solution concentrations and immersion times were the following:

(1) Immersion in Santa Fe municipal water during storage and shipping;
(2) Immersion in 2.5% gluteraldehyde-water solution overnight;
(3) Immersion in 25% ethanol-water solution overnight;
(4) Immersion in 50% ethanol-water solution overnight;
(5) Immersion in 75% ethanol-water solution overnight;
(6) Immersion in 95% ethanol-water solution overnight;
(7) Immersion in 100% ethanol-water solution overnight;
(8) Immersion in 33% HMDS–ethanol solution for 10 min;
(9) Immersion in 50% HMDS–ethanol solution for 10 min;
(10) Immersion in 100% HMDS–ethanol solution for 10 min;
(11) Dry overnight in a desiccant chamber;
(12) Platinum sputter coat for viewing in SEM.

## 4. Conclusions

SEM investigation has shown the presence of microbes inside corrosion pits in copper tubing. These microbes are proposed to be responsible for the corrosion pitting, and they visually resemble Actinobacteria. The authors propose that the microbes gain a foothold on copper surfaces due to the presence of residual organic films from the manufacturing process. Once established, the microbes form the pit/membrane/mound system and slowly dissolve the underlying copper. If other cases of pitting in copper tubing were caused by microbiologically induced corrosion (MIC), then prevention and eradication should be possible with different surface preparation techniques, and with any of several different methods that can alter environmental conditions and kill the microbes or inhibit their establishment and growth. 
